# Epigenetic Drugs Can Stimulate Metastasis through Enhanced Expression of the Pro-Metastatic Ezrin Gene

**DOI:** 10.1371/journal.pone.0012710

**Published:** 2010-09-13

**Authors:** Yanlin Yu, Pingyao Zeng, Jingbo Xiong, Ziyang Liu, Shelley L. Berger, Glenn Merlino

**Affiliations:** 1 Laboratory of Cancer Biology and Genetics, National Cancer Institute, National Institutes of Health, Bethesda, Maryland, United States of America; 2 Department of Cell and Developmental Biology, University of Pennsylvania, Philadelphia, Pennsylvania, United States of America; 3 Department of Cell Biology, Southern Medical University, Guangzhou, Guangdong, People's Republic of China; Roswell Park Cancer Institute, United States of America

## Abstract

Ezrin has been reported to be upregulated in many tumors and to participate in metastatic progression. No study has addressed epigenetic modification in the regulation of Ezrin gene expression, the importance of which is unknown. Here, we report that highly metastatic rhabdomyosarcoma (RMS) cells with high levels of Ezrin have elevated acetyl-H3-K9 and tri-methyl-H3-K4 as well as reduced DNA methylation at the Ezrin gene promoter. Conversely, poorly metastatic RMS cells with low levels of Ezrin have reduced acetyl-H3-K9 and elevated methylation. Thus epigenetic covalent modifications to histones within nucleosomes of the Ezrin gene promoter are linked to Ezrin expression, which in fact can be regulated by epigenetic mechanisms. Notably, treatment with histone deacetylase (HDAC) inhibitors or DNA demethylating agents could restore Ezrin expression and stimulate the metastatic potential of poorly metastatic RMS cells characterized by low Ezrin levels. However, the ability of epigenetic drugs to stimulate metastasis in RMS cells was inhibited by expression of an Ezrin-specific shRNA. Our data demonstrate the potential risk associated with clinical application of broadly acting covalent epigenetic modifiers, and highlight the value of combination therapies that include agents specifically targeting potent pro-metastatic genes.

## Introduction

Tumor genesis and progression to metastasis are fueled through dysregulation of genes and/or signaling pathways resulting in abnormal cell functions and behaviors [1]–[3]. Ezrin has been reported to be upregulated in many tumors, where it can promote the metastatic phenotype [4]–[6]. In particular, Ezrin was determined to be a critical regulator of metastasis in pediatric sarcomas such as rhabdomyosarcoma (RMS) and osteosarcoma [7]–[9]. Ectopic expression of Ezrin in poorly metastatic cells enhanced metastasis, whereas downregulation of endogenous Ezrin in highly metastatic cells inhibited metastasis [7]. Ezrin has also been implicated in the metastasis of breast cancer [10], [11], pancreatic adenocarcinoma [12], osterosarcoma [8], [9], melanoma [13], [14] and prostate cancer [15].

Ezrin, encoded by *Vil2*, was identified 20 years ago as the first member of the ERM family (Ezrin-Radixin-Moesin) within the band 4.1 super family [16], [17]. Ezrin is a physical and functional linker between the plasma membrane and the actin cytoskeleton and an organizer of membrane-cytoskeleton–associated complexes. As such, Ezrin is known to mediate multiple cellular activities including survival, adhesion and migration/invasion [16]–[18], thereby regulating tumor development and progression through signal transduction pathways involving protein kinase A, Rho, phosphatidylinositol 3-kinase/Akt, mitogen-activated protein kinase (MAPK) and Src [16]–[20]. Although the function of Ezrin is well studied, the transcriptional regulation of Ezrin is poorly understood.

The process of gene transcription is controlled through orchestration of myriad transcription factors and epigenetic modifications. Our previous study showed that the homeoprotein transcription factor Six1 could bind to the mouse Ezrin gene (*vil2*) promoter and regulate its transcription [21]. A recent study proposed that transcriptional factors Sp1 and AP-1 might regulate expression of the human *Vil2* gene in esophageal carcinoma cells [22]. However, no study has addressed the importance of epigenetic modification in the regulation of Ezrin gene expression.

Unlike transcription factors, which physically and transiently bind to gene promoter regions and function in the process of transcription [23], epigenetic modulations of the genome involving histone modifications and DNA methylation at gene promoter regions, altering the gene chromatin configuration. A decondensed (‘open’) configuration allows DNA binding proteins such as transcription factors access to binding sites, whereas a condensed (‘closed’) configuration blocks transcription binding sites, thereby regulating gene transcription [24]. Ample evidence suggests that epigenetic mechanisms play a significant role in the development and progression of tumorigenesis. Epigenetic changes such as acetylation, deacetylation and methylation of chromatin histone protein and DNA methylation result in the alteration of gene expression [25], [26]. Chromatin histone acetylation by histone acetytransferase (HAT), deacetylation by histone deacetylase (HDAC) and methylation by histone lysine methytransferases (HMT) can alter chromatin structure and dynamically affect transcriptional regulation [24]. In general, acetylation of core histone lysine by HAT has been associated with increased gene transcription, whereas deacetylation of core histone lysine by HDAC has been related to decreased gene transcription; for example, acetylated histone H3 lysine 9 (acetyl-H3-K9) is frequently associated with gene activity [25]. In contrast, histone lysine methylation can result in either activation or repression, depending on the residue on which it resides. Histone H3 lysine 4 (H3-K4) methylation is a well-known ‘active’ marker, but methylation of histone H3 lysine 9 (H3-K9) is a marker of gene inactivity [25], [26]. Associated with histone modification, DNA methylation regulated by DNA methytransferase (DNMTs) at the cis-regulatory region (CpG islands) of genes also acts as an epigenetic switch to turn gene expression on or off. When DNA is methylated in the promoter region of genes, where transcription is initiated, they are typically inactivated and silenced [27]–[29].

In the current study, we examined the status of histone modification and DNA methylation at the Ezrin gene locus in highly and poorly metastatic RMS cell lines. We found that RMS cells with elevated Ezrin expression and high metastatic potential had greater acetylation of histone H3 lysine 9 (acetyl-H3-K9) and tri-methylation of histone H3 lysine 4 (tri-methyl-H3-K4). In contrast, RMS cells with low Ezrin expression and poor metastatic potential had diminished levels of acetyl-H3-K9 and tri-methyl-H3-K4 instead of high levels of di-methylation of histone H3 lysine 9 (di-methyl-H3-K9). The status of DNA methylation at the Ezrin gene promoter region correlated with histone modification and Ezrin expression. Treatment with inhibitors of histone deacetylase (HDACis) and DNA methylation restored (or upregulated) expression of Ezrin and enhanced metastatic behavior. Our data demonstrate for the first time that epigenetic covalent modifications to histones within nucleosomes of the Ezrin gene promoter are linked to Ezrin expression, and hence to metastastic behavior.

## Results

### Epigenetic modifications at the Ezrin gene locus are linked to its expression

**Figure 1:**
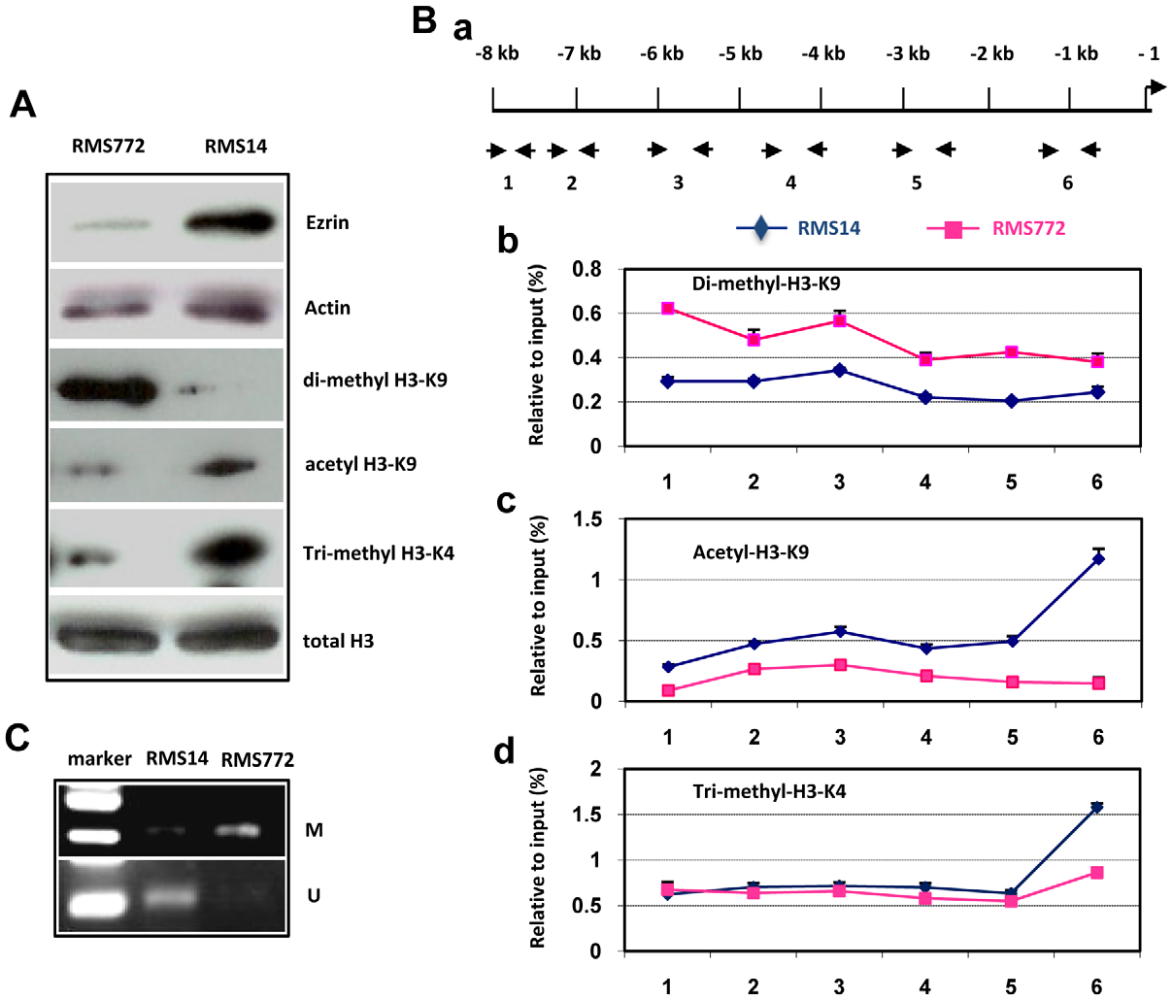
Epigenetic modifications at the Ezrin gene locus are linked to its expression. (A) Comparative expression and activity of histone H3 tail proteins were analyzed by Western blotting in RMS772 and RMS14 cells. RMS772 cells with low level Ezrin have a higher level of di-methyl-H3K9, but lower levels of actyl-H3K9 and tri-methyl-H3K4; in contrast, RMS14 cells with higher level Ezrin have higher levels of acetyl-H3K9 and tri-methyl-H3K4, but a lower level di-methyl-H3K9. (B) Comparative analysis of histone modifications at the Ezrin gene promoter was performed by chromatin immunoprecipitation (ChIP) in RMS772 cells (purple line) and RMS14 cells (blue line). Six pairs of primers were designed for covering an 8 kb region of the Ezrin gene promoter (a). Enriched chromatin DNA immunoprecipitated with anti-di-methyl-H3K9 (b), anti-acetyl-H3K9 (c) or anti-tri-methyl-H3K4 (d) were amplified by PCR using six pairs of primers. The data were analyzed using Image J software and normalized with input. Purple line represents data from RMS772 cells; blue line represents data from RMS14 cells. (C) The status of DNA methylation within the Ezrin gene promoter was analyzed by methylation-specific PCR using specific methylated Ezrin promoter primers and specific unmethylated Ezrin promoter primers. M, methylated promoter; U, unmethylated promoter.

We previously reported that Ezrin expression was significantly increased in highly metastatic mouse RMS cell lines compared to poorly metastatic cell lines [7], [21], but the mechanism by which the Ezrin gene is regulated, and the role of epigenetic modification, remain mostly unknown. To explore these mechanisms, we evaluated the covalent modifications within the histone H3 tails of the Ezrin gene by western blotting. Two mouse RMS cell lines, RMS772 and RMS14, were used for comparative analysis. RMS772 cells have very low or undetectable expression of Ezrin and poor metastatic potential in vivo; RMS14 cells have a high level of Ezrin protein and high metastatic potential [7], [21]. As shown in Figure 1A, high-Ezrin RMS14 cells have an elevated level of acetylation of histone H3 lysine 9 (acetyl-H3-K9) and tri-methylation of histone H3 lysine 4 (tri-methyl-H3-K4), but a lower level of di-methylation of histone lysine 9 (di-methyl-H3-K9). In contrast, low-Ezrin RMS772 cells have reduced actyl-H3-K9 and tri-methyl-H3-K4, but a notably higher level of di-methyl-H3-K9.

This finding led us to hypothesize that chromatin/histone modifications help regulate Ezrin gene transcription. To confirm this, we performed ChIP assays to analyze histone modifications across an 8 kb region of the Ezrin gene promoter in RMS14 and RMS772 cells using 6 paired primers (Figure 1B-a). The results show that acetylated and/or methylated histone H3 had different patterns of enrichment within the Ezrin gene promoter in the two RMS cell lines. Di-methyl-H3-K9 was enriched along the Ezrin gene promoter (across 8 kb) in RMS772 cells (Figure 1B-b). In contrast, acetylated-H3-K9 was relatively enriched along the entire hyperacetylated region in RMS14 cells (Figure 1B-c). Tri-methyl-H3-K4 appeared to be enriched around the transcription start site in RMS14 cells relative to RMS772 cells, although no difference was observed in the other regions (Figure 1B-d). Historically, di-methyl-H3-K9 enrichment has been associated with gene silencing, and acetyl-H3-K9 and tri-methyl-H3-K4 with gene activation [24], [25]. These data are consistent with our previous results, which showed that Ezrin expression levels are higher in RMS14 than in RMS772 (Figure 1A), suggesting that Ezrin expression correlates with histone H3 tail modification.

Beside histone modification, another epigenetic mechanism by which gene transcription can be regulated is through promoter DNA methylation. Promoter hypermethylation is known to silence gene expression, whereas hypomethylated promoters are generally transcriptionally active [29]. Therefore, we next used methylation-specific PCR to compare methylated DNA and unmethylated DNA in the Ezrin gene promoter in RMS14 cells and RMS772 cells. We found that the Ezrin gene promoter in RMS14 cells was unmethylated, whereas in RMS772 cells the Ezrin promoter was overtly methylated (Figure 1C). These results indicate that histone and DNA modifications at the Ezrin gene promoter locus correlate with the expression level of Ezrin, and suggest that these modifications regulate Ezrin gene expression.

### HDAC inhibitors and demethylating agents reactivate Ezrin gene expression

**Figure 2:**
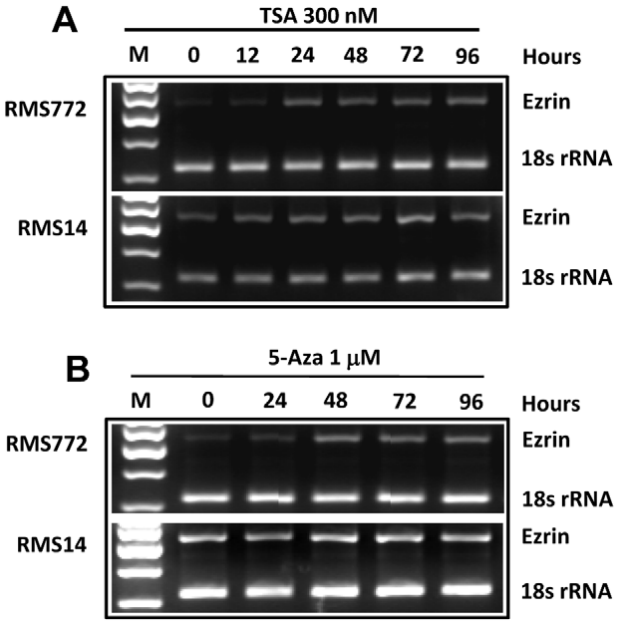
HDAC inhibitor (TSA) and DNA demethylating agent (5-Aza) reactivate Ezrin gene expression. The expression of Ezrin was analyzed by RT-PCR after treatment with 300 nM TSA or 1 µM 5-Aza in RMS772 and RMS14 cells. (A) and (B) Ezrin expression in different time courses after treatment with TSA (A) or 5-Aza (B) in RMS772 and RMS14 cells. M, DNA molecular marker; 18s rRNA, internal controls.

Epigenetic changes are known to be reversible. HDACis such as trichostatin A (TSA) can increase expression of genes by inhibiting histone deactylase activity. Inhibitors of DNA methylation such as 5-aza-2′-deoxcytidine (5-Aza) can demethylate promoter regions and reactivate silenced genes, restoring their function [30]. To determine if the epigenetic changes we detected could be further linked to Ezrin expression, we examined Ezrin expression by qRT-PCR following treatment with either TSA or 5-Aza. After a 24-hour treatment with 300 nM TSA, the level of Ezrin expression was increased in RMS772 cells (Figure 2A); a similar result was found after a 48-hour treatment with 1 µM 5-Aza in RMS772 cells (Figure 2B). Notably, these drugs did not affect Ezrin expression in RMS14 cells, where the Ezrin gene is already highly expressed (Figure 2A and B) and its chromatin characterized by high histone acetylation and low DNA methylation (Figure 1). These results show that both the HDACi TSA and the DNA demethylating agent 5-Aza can reactivate Ezrin expression, and link modifications at the Ezrin gene locus with its expression.

### Epigenetic agents can regulate metastatic potential through modulation of Ezrin gene expression

**Figure 3:**
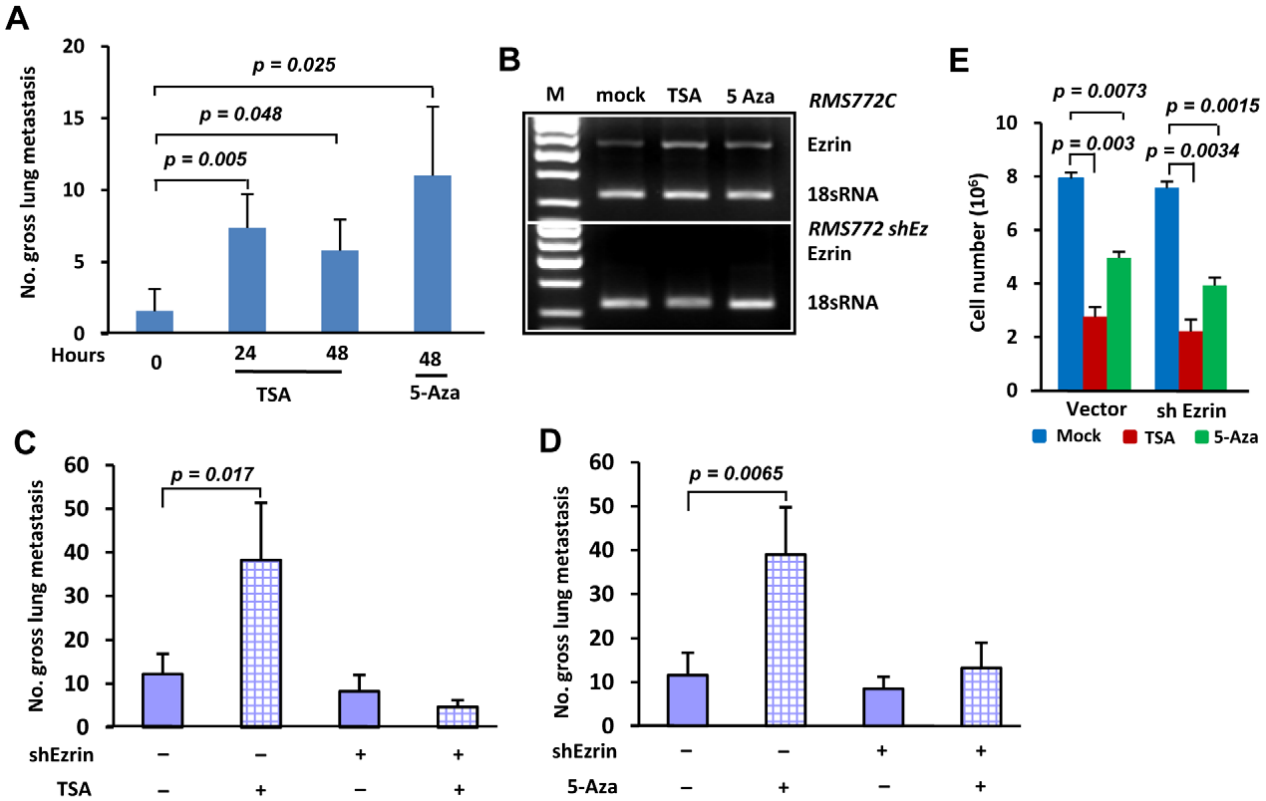
Epigenetic agents regulate metastatic potential in pretreated cultured RMS772 cells. (A) Gross pulmonary metastases from pretreated cultured RMS772 cell with either 300 nM TSA (24 or 48 hours) or 1 µM 5-Aza (48 hours) in cell culture. After pretreated with epigenetic agents, 2×10^5^ cells were injected into athymic nude mice by tail vein. (B) Ezrin expression after treatment with TSA and 5-Aza for 48 hours in RMS772 cells with stably transfected shRNA Ezrin expression vector (RMS772 shEz) or empty vector (RMS772C). M, DNA molecular marker; 18s rRNA, internal controls. (C) and (D) Gross pulmonary metastases from cells with stably transfected shRNA Ezrin expression vector (+) or empty vector (−) pretreated with either 300 nM TSA (C) or 1 µM 5-Aza (D) for 48 hours in cell culture. After pretreated with epigenetic agents, 5×10^5^ cells were injected into athymic nude mice by tail vein. (E) Cell growth was represented by the number of cells. RMS772 cells with stably transfected shRNA Ezrin expression vector (shEzrin) or empty vector (vector) were treated with 300 nM TSA (red rectangle), 1 µM 5-Aza (green rectangle) or mock DMSO (Blue rectangle) for 48 hours.

We and others have reported that Ezrin is a critical regulator of metastasis in RMS and osteosarcoma [7]–[9], and our data now show that HDACis and DNA demethylating agents can restore Ezrin expression. Many HDACis as well as DNA demethylating agents are currently in clinical trials as anticancer drugs [31], [32]. This raises the disturbing possibility that epigenetic drugs may be capable of enhancing metastasis. To address this question, we first pretreated cultured RMS772 cells with either TSA or 5-Aza, then introduced those cells into athymic nude mice by tail vein injection. The metastatic ability of RMS772 cells pretreated with either drug was elevated significantly compared with untreated cells (Figure 3A). To determine if epigenetic drug-stimulated metastasis is associated with Ezrin expression, a vector expressing Ezrin shRNA and GFP was introduced into poorly metastatic, low-Ezrin RMS772 cells. Pools of shRNA-GFP positive cells, purified by three rounds of GFP-based FACS, were used for experimental metastasis. Ezrin shRNA successfully down-regulated endogenous Ezrin, and also abrogated the TSA- and 5-Aza-enhanced Ezrin expression (Figure 3B). After pretreatment with TSA or 5-Aza for 48 hours in culture, the transfected RMS772 cells were injected into athymic nude mice and their metastatic potential determined. Both drugs again significantly stimulated the pulmonary metastasis of control RMS772 cells; however, their ability to stimulate metastasis was now inhibited by expression of Ezrin shRNA (Figure 3C, D).

**Figure 4:**
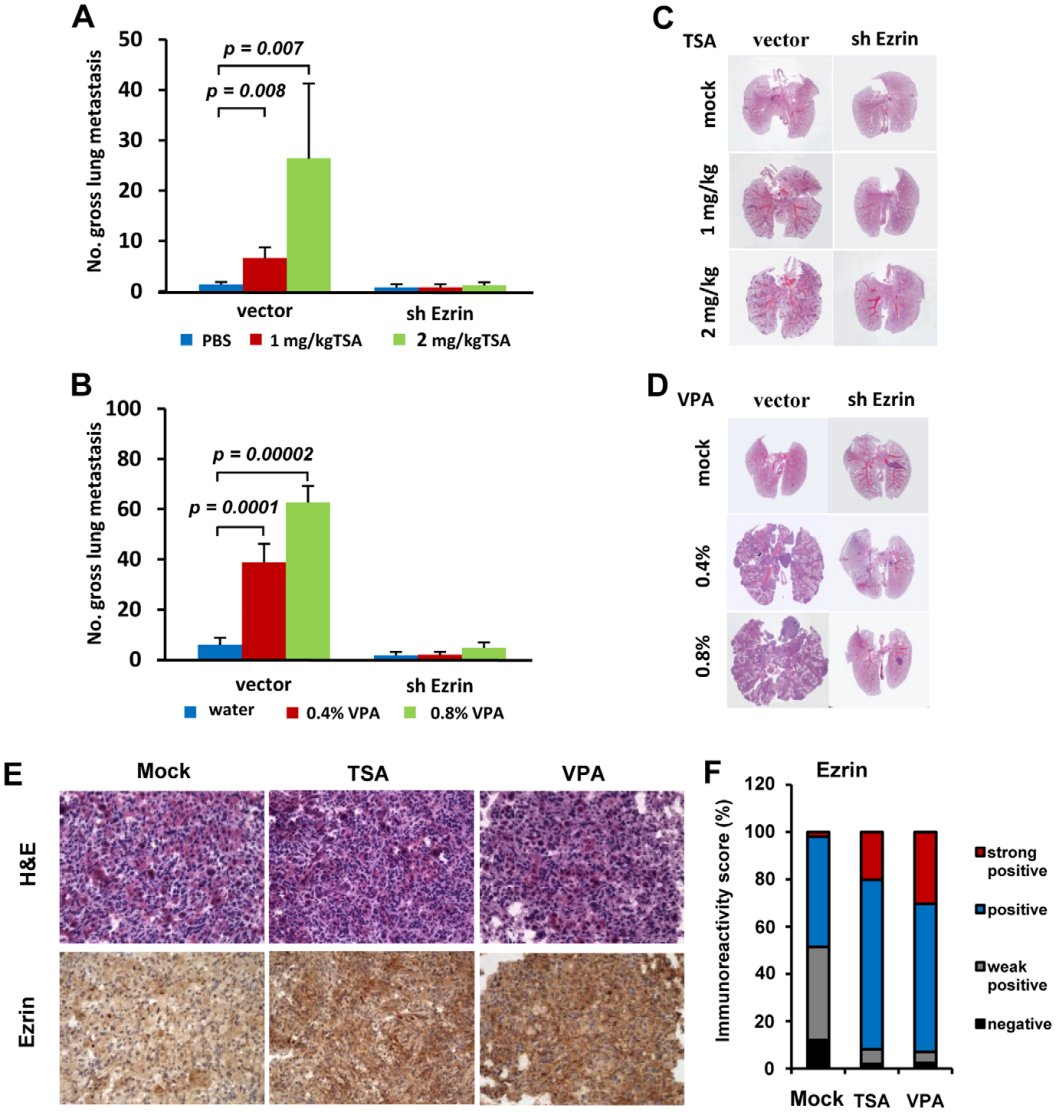
Epigenetic agents regulate metastatic potential in preclinical mice model. (A) and (B) Gross pulmonary metastases from mice treated with TSA (A) or VPA (B). Mice harboring 5×10^5^ RMS772 cells stably transfected with the shRNA Ezrin expression vector (shEzrin) or empty vector (vector) were treated with two different doses of TSA or VPA for four days. (C) and (D) Representative histopathology (H&E staining) of lung sections with metastases from mice treated with TSA (C) or 5-Aza (D). Vector, lung section from mouse inoculated with RMS772 cells with empty vector control; shEzrin, lung section from mouse inoculated with RMS772 transfected with the shRNA Ezrin expressing vector. (E) Ezrin expression as determined by immunohistochemical staining in metastases derived from mice bearing RMS772 cells treated with 2 mg/kg TSA (TSA) or 0.8% VPA (VPA). H&E, hematoxylin and eosin staining; Ezrin, immunohistochemical staining with anti-Ezrin antibody; 20X. (F) Immunoreactivity score of Ezrin expression in metastases from mice treated with 2 mg/kg TSA (TSA) or 0.8% VPA (VPA). Quantitative scores, which were analyzed using ImageScope V10.0 software from Aperio Technololgies, presenting the percentage of cells in strongly positive, positive, weakly positive and negative for immunohistochemical staining with an anti-Ezrin antibody.

To evaluate the effects of epigenetic drugs on metastasis in a more relevant preclinical mice model, we designed and performed animal studies focusing on the two HDACis TSA and valproic acid (VPA). Immediately following injection of parental RMS772 cells into athymic nude mice, two different doses of each drug were administered to mice over the next four days either by intraperitoneal injection for TSA (administered once per day), or in the drinking water for VPA. Mice injected with parental RMS772 cells and treated with either of the HDACis bore a significantly higher number of pulmonary metastases (Figure 4A, B, C, D left panels). However, mice injected with RMS772 cells that had been transfected with shRNA Ezrin developed significantly fewer metastases despite the presence of either HDACi, essentially the same number as the untreated control group (Figure 4A, B, C, D right panels). Immunohistochemical staining showed that Ezrin expression in the metastases derived from mice treated with HDACis was more intense (Figure 4E) and had an increased percentage of strong positive cells (mock 1.96%, TSA 20.18% and VPA 30.33%) and positive cells (mock 46.62%, TSA 71.76%, and VPA 62.62%) (Figure 4F), indicating that the HDACis had increased Ezrin expression in this preclinical mouse model. Taken together, our data indicate that HDACi treatment can actually enhance the metastatic potential of RMS in vivo, and that this increase is mediated by epigenetic deregulation of Ezrin gene expression.

**Figure 5:**
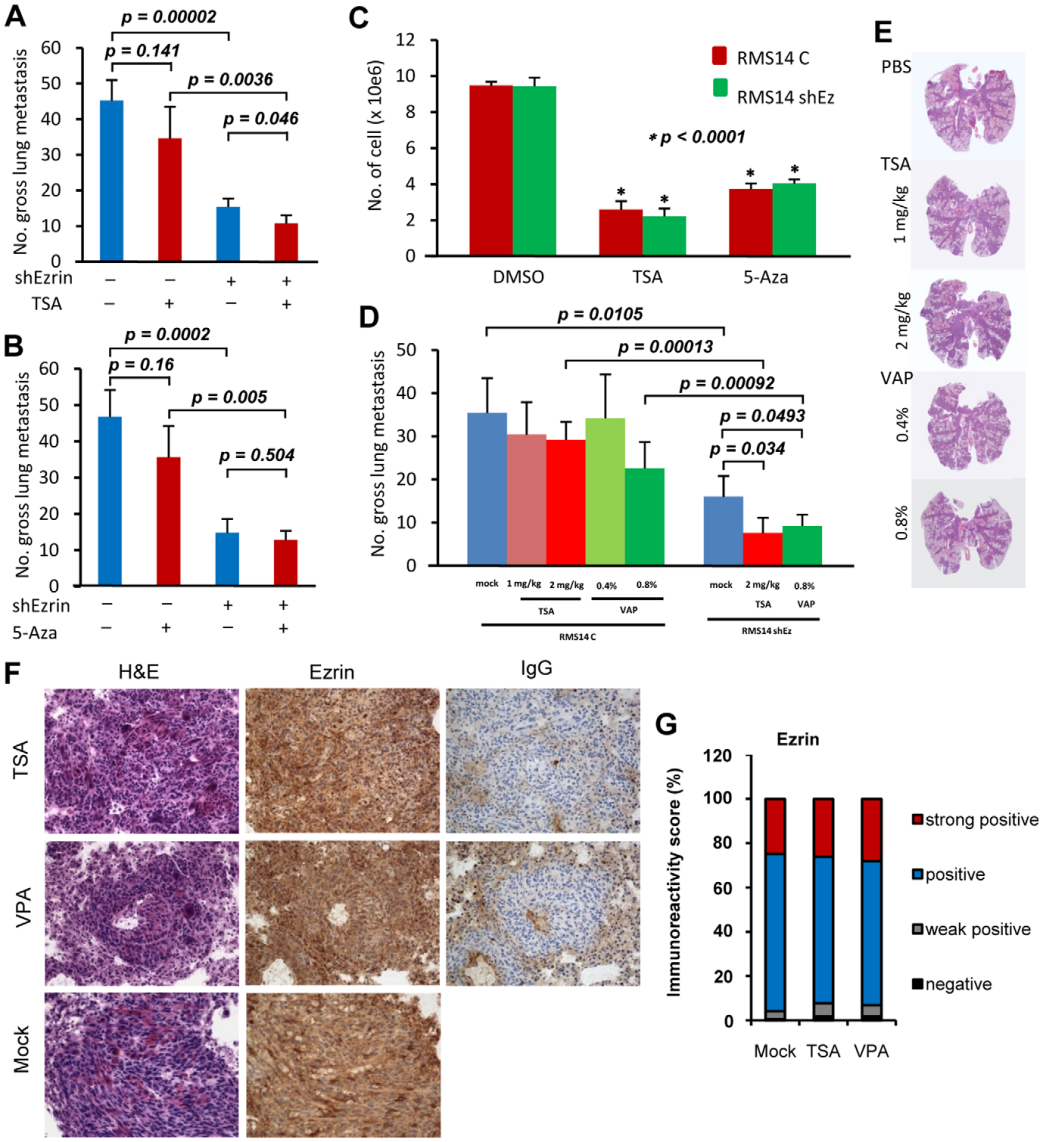
Phenotypic effects of epigenetic drugs on RMS14 cells. RMS14 cells with high-level endogenous Ezrin have high metastatic potential. (A) and (B) Gross pulmonary metastases from RMS14 cells stably transfected with an shRNA Ezrin expression vector (+) or empty vector (−), pretreated with 300 nM TSA (A) or 1 µM 5-Aza (B) for 48 hours in cell culture. (C) Growth rate of RMS14 cells treated with TSA or 5-Aza stably transfected with either shRNA Ezrin expression vector (RMS14 shEz) or empty vector (RMS14C). There was no statistic difference in cell number between RMS14C and RMS14 shEz in the DMSO group, but treatment with either TSA or 5-Aza significantly inhibited cell growth in both RMS14C and RMS14 shEz cells (*p<0.0001). (D) Gross pulmonary metastases from mice treated with TSA or VPA. Mice harboring RMS14 cells stably transfected with either the shRNA Ezrin expression vector (RMS14 shEz) or empty vector (RMS14C) were treated with TSA or VPA for four days. (E) Representative histopathology (H&E staining) of lung sections with metastases from mice bearing RMS14C and RMS14 shEz cells treated with TSA or VPA. (F) Ezrin expression assessed by immunohistochemical staining in metastases derived from mice treated with 2 mg/kg TSA or 0.8% VPA; H&E, hematoxylin and eosin staining; Ezrin, immunohistochemistry staining with anti-Ezrin antibody; IgG, negative control. 20X. (G) Immunoreactivity score of Ezrin expression in metastases from mice treated with 2 mg/kg TSA or 0.8% VPA. Ezrin expression in tissue sections was visualized using an anti-Ezrin antibody, and quantified using ImageScope V10.0 software from Aperio Technololgies; the percentage of cells that were strongly positive, positive, weakly positive or negative for Ezrin is presented.

Studies have shown that epigenetic drugs can affect a number of tumor cell phenotypes, including proliferation [31]. Interestingly, despite their ability to enhance metastasis, we discovered that the number of cultured RMS772 cells was significantly reduced when exposed to either TSA or 5-Aza (Figure 3E). These epigenetic agents also reduced the number of cultured metastatic RMS14 cells (Figure 5C). Expression of Ezrin shRNA did not alter the ability of TSA or 5-Aza to influence cell growth (Figure 3E and Figure 5C), indicating that this ability of these epigenetic drugs is independent of Ezrin.

We have demonstrated the risk of epigenetic drug treatment on otherwise poorly metastatic tumor cells in which potent pro-metastatic genes such as Ezrin can be epigenetically upregulated. What then are the consequences in cells that are already highly metastatic, such as high-Ezrin RMS14? Neither TSA nor 5-Aza overtly affected Ezrin expression in metastatic high-Ezrin RMS14 cells (Figure 2A, B). Pretreatment of cultured RMS14 cells with either TSA or 5-Aza did not affect metastasis significantly (Figure 5A and B), although the number of metastases was reduced. Similarly, no significant difference in metastasis of parental RMS14 cells was found in mice treated by either TSA or VPA (Figure 5D, E). Consistent with in vitro results in which TSA and 5-Aza failed to affect Ezrin expression in high-Ezrin RMS14 cells (Figure 2A and B), immunostaining showed that these drugs did not overtly affect Ezrin expression in RMS14 cells in vivo (Figure 5F and G). However, the HDACis had a more significant effect on RMS14 cell metastasis when Ezrin gene expression was simultaneously reduced by the Ezrin-specific shRNA (Figure 5A and D). As in RMS772 cells, TSA and 5-Aza were able to significantly inhibit RMS14 cell growth in vitro (Figure 5C). Taken together, data from our model reveal that the potential for epigenetic drugs to stimulate metastasis correlates with its ability to induce expression of potent pro-metastasis genes, and suggests that the inhibition of pro-metastasis genes such as Ezrin can enhance, or even be required for, the efficacy of epigenetic drugs in treating RMS patients.

## Discussion

Epigenetics is a rapidly evolving field that attempts to explain how heritable changes in gene expression occur without altering nucleotide sequence [33]. Increasing evidence now indicates that epigenetic regulation plays a crucial role in the regulation of gene expression [34], [35]. Here, we report for the first time that Ezrin, which links the plasma membrane to the cytoskeleton [16], [17], is regulated by epigenetic modifications including histone modifications and DNA methylation to the promoter region. Upregulation of Ezrin gene expression is associated with the histone ‘active code’ (acetyl-H3-K9 and tri-methyl-H3-K4) and with unmethylated CpG islands within the Ezrin promoter; in contrast, downregulation of Ezrin is linked to the histone ‘repressive code’ (di-methyl-H3-K9) and with methylated CpG islands. HDACis such as TSA and DNMT inhibitors such as 5-Aza are well known epigenetic drugs [30]–[32]. We show that treatment with either TSA or 5-Aza enhances Ezrin levels in cells characterized by low Ezrin expression such as RMS772 cells, further corroborating the epigenetic regulation of Ezrin.

Ezrin, whose expression correlates with progression in many tumor types, is involved in multiple metastatic pathways [16]–[18]. For example, Ezrin interacts with the cell surface receptor CD44 to promote invasiveness [36]–[38], and incites metastasis via Rho activation [7], [39]. Ezrin plays a role in metastasis-associated cell-adhesion functions through interactions with E-cadherin [40]. Ezrin also influences cell adhesion and migration as a downstream target of Src [20], [41], and as a direct target of MET, a receptor tyrosine kinase frequently implicated in metastatic behavior [42], [43]. We previously reported that Ezrin is a direct transcriptional target of the homeoprotein Six1, and the predominant mediator of Six1-stimulated metastasis [21]. Our findings in this study that epigenetic modifiers can upregulate Ezrin expression prompted us to explore the functional consequences of exposure to HDACi and demethylation agents on metastasis.

Based on their ability to reactivate tumor suppressor genes silenced by DNA methylation and chromatin modification, DNA methylation inhibitors and HDACis are emerging as a new class of anticancer agents and are in many ongoing patient treatment regimens [30]–[32], [44]–[46]. Tumor suppressor genes reactivated by epigenetic agents include p53, p16, p21 and PTEN, thereby inhibiting cell proliferation, survival, angiogenesis, cell migration and metastasis [46]–[49]. Consistent with these reports, our data suggest that TSA and 5-Aza can both inhibit cell proliferation. However, their effect on Ezrin gene expression in tumor cells raised the possibility that metastatic potential could none-the-less be enhanced, a possibility that we confirm in this report; the ability of epigenetic drugs to stimulate metastasis in our RMS cell lines was found to be associated with Ezrin expression.

It is worth noting that this is likely not a RMS-specific phenomenon. These same drugs were also able to increase Ezrin expression and enhance the metastatic potential of human melanoma A375 cells, while inhibiting cell growth (Supplementary Figure S1).

However, neither epigenetic drug significantly affected Ezrin expression in metastatic high-Ezrin RMS14 cells; moreover, their influence on RMS14 metastasis was minimal. This is likely due in large part to the fact that the Ezrin gene locus in RMS14 cells is already epigenetically configured for high Ezrin expression. Regardless, both TSA and 5-Aza “worked” in RMS14 cells, dramatically inhibiting cell growth in an Ezrin-independent fashion. Interestingly, when Ezrin gene expression was reduced (i.e., using forced expression of Ezrin-specific shRNA; 7, 21), the combination of HDACi then had an inhibitory effect on metastasis. Our findings suggested that the ability of TSA or 5-aza to stimulate metastasis correlates with its ability to induce Ezrin expression.

Our data support the notion that anticancer agents can drive tumor promotion and metastasis [50], [51], and that an epigenetic drug can be a double-edged sword. Epigenetic drugs can affect many genes and multiple pathways, including some capable of inducing cancer cell migration and invasion, such as CCR7, CXCR4, uPA and ROS [52]–[55]. Clearly, the well-known ability of epigenetic drugs to block proliferation is not sufficient to inhibit metastatic behavior, at least in the RMS cells we tested. This finding may help to explain the not infrequent failure of these promising agents in the clinic.

In summary, three key points of our work are of clinical relevance with respect to metastasis. First, expression of pro-metastatic Ezrin can be regulated by epigenetic modification, thus representing a model for assessing the relationship between epigenetic modification and metastasis. Second, commonly employed epigenetic modifiers can restore Ezrin expression, and with it metastatic potential, highlighting the potentially adverse effects on specific patient populations. Third, treatment using such epigenetic drugs can still be effective if expression of pro-metastatic genes such as Ezrin gene is controlled. Our data suggest that the potential for epigenetic drugs to stimulate metastasis in the clinic correlates with its ability to induce expression of potent pro-metastasis genes, and that simultaneous inhibition of these genes, such as Ezrin, can enhance or even be required for the clinical efficacy of epigenetic drugs. We suggest that Ezrin might represent a new class of prognostic markers for assessing the metastatic risk of epigenetic drugs, and a novel therapeutic target for developing more efficacious combination treatment strategies.

## Methods

### shRNA plasmids and antibodies

Three shRNA expressing plasmids were used in this study: one was constructed in the pSUPER vector employing synthesized double stranded DNA fragments directed against nucleotides 174 to 194 of the mouse ezrin (XM-123004) coding region [7], [21], using pSUPER as control; the other two were based on pGIPZ (NM_009510), purchased from Open biosystems (Huntsville, AL). Antibodies used included: anti-Ezrin (Sigma, St. Louis, MO); anti-histone H3, anti-tri-methtl-histone H3 (Lys4), anti-di-methyl-histone H3 (Lys9), anti-acetylaed histone H3 (Lys9) (Upstate, NY); anti-β-actin (Santa Cruz Biotechnology Inc, Santa Cruz, CA).

### Cell lines and cell culture

RMS772 and RMS14 cell lines were derived from RMS tumors arising in HGF/SF-transgenic, Ink4a/Arf-deficient mice [7]. RMS14 shEz cell line and RMS14C cell line were establised in our previous study by stably transfected with either pSUPER shRNA Ezrin or pSUPER empty vector [7]. RMS772 was characterized by low Ezrin levels [7]. The stable shRNA-expressing RMS772 cells were established through transfection of pGIPZ shEzrin or pGIPZ empty vector and sorting by Fluorescence Activated Cell Sorting (FACS). All RMS cell lines were maintained in RPMI1640 medium supplemented with 10% Fetal Bovine Serum (FBS) (Invitrogen, Carlsbad, CA); A375 was a gift from Dr. Isaiah Fidler at M.D. Medical Center (Houston, Texas) and maintained in EME (Earle's) with 10% FBS, 2 mM L-glutamine, 2 x Vitamins, non-essential amino acids, 1 mM sodium pyruvate.

### Western blot

For detection of histone proteins, the acid extraction of protein from cells (acid-extracted total protein from log phase cells) was performed according to the following protocol. Briefly, cells were grown to 70–85% confluency, collected and lysated in 5–10 volumes of lysis buffer (10 mM HEPES, pH 7.9; 1.5 mM MgCl_2_; 10 mM KCl; 0.5 mMDTT and 1.5 mM PMSF). HCl was added to the cell lysate to a final concentration of 0.2 M. After 30 minutes of incubation on ice, the supernatant fraction containing the acid soluble proteins was collected by centrifugation at 11,000 g for 10 minutes at 4°C. The supernatant was dialyzed against 200 ml 0.1 M acetic acid twice for 2 hours each, and three times against 200 ml water for 1 hour, 3 hours and overnight, respectively. The dialyzed supernatant was collected for western blot analysis. For detection of Ezrin and actin expression, see reference [21], [56].

### Epigenetic drugs treatments

Cells were treated with mock (DMSO), 300 nM Trichostatin A (TSA, Sigma, St. Louis, MO) or 1 µM 5-Aza-2′ deoxycytidine (5-Aza, Sigma, St. Louis, MO) for 12, 24, 48, 72, 96 hrs. Animals used were male athymic nude mice between 4 and 6 weeks of age. After being subjected to intraveneous injection via the tail vein with cultured cells, all mice were treated for 4 days with either TSA (1 mg/kg or 2 mg/kg) by injecting i.p. daily, or Valproic acid (VPA, Sigma, St. Louis, MO) (0.4% or 0.8%) in drinking water, and then put on regular water for the duration of the experiment. All animals had to be held for between 3 weeks post injection to achieve valid metastasis analyses. All mouse procedures were performed according to NIH guidelines [the animal proposal: LMB-052, approval by NCI-Bethesda Animal Care and Use Committee (ACUC)].

### Chromatin immunoprecipitation (ChIP) assays

ChIP assays were performed as previous described [21]. Briefly, cells were formaldehyde cross-linked for 15 min at room temperature (RT) by adding 0.1 volumes of cross-linking solution directly to the culture medium in the plates. Cross-linking was stopped by the addition of glycine to a final concentration of 125 µmol/L. Cells were washed twice with ice-cold PBS, harvested in PBS by scraping, and subjected to ChIP analysis following the Chromatin Immunoprecipitation Assay Kit manufacturer's instructions (Upstate, NY). Immunoprecipitated DNAs were analyzed by PCR using the different Ezrin promoter primers: 1, ctg cga aca ccc taa act ac and ctg act gaa gca aga acc ac; 2, tct cca gcc cca act ttt atc and aca tcc cac cca tct gtc tc; 3, tgc cac atc ctt gtc tgt c and ctc att aac cct gta gct gtc; 4, cct aga aaa cct cga aac aca c and act cgc tcc tat ttg ctc c; 5, tgc ctc atc tcc tta tcc cc and tcc tct agt cta tca acc ccc; 6, tcc agg cat ctg agg aat ac and gat cca agg agc aac atc tac. The data were analyzed using Image J software (NIH) and normilized with input.

### Relative quantitative reverse transcriptase polymerase chain reaction

Total RNA was extracted from cells using TRIzol Reagent (Invitrogen, Carlsbad, CA). RNA concentration, purity, and integrity were determined by UV spectrophotometry. Two µg of total RNA were incubated with 30 ng random primer at 42°C for 30 min in a final volume of 20 µl reaction mixture containing 1 x reaction buffer, 5 mmol/L dNTP, 10 mmol/L DTT, 0.5 U/µl RNasin (Promega, Madison WI), and 200 U superscript RNase H-M-MLV reverse transcriptase (Invitrogen, Carlsbad, CA), and the reaction mixtures incubated at 95°C for 10 min. One-µl reaction mixtures were amplified in 25 µl PCR reaction mixtures containing 1 x PCR reaction buffer, 1.5 mmol/L MgCl_2_, 100 µmol/L dNTP, 5 pmol primers, 1 µl 18S rRNA primer set (Ambion, Austin, TX) as internal standards and 1 U Taq DNA polymerase (Invitrogen, Carlsbad, CA) for 30 cycles at 94°C, 30 seconds; 55°C, 30 seconds; and 72°C, 30 seconds. Following PCR, 10 µl of the reaction were run in a 2% agarose gel, the PCR bands imaged using EAGLE EYE II (Stratagene, La Jolla, CA), and the data analyzed using Image J software (NIH). The sense and antisense primers used for mouse Ezrin were aca gag gca gag aag aat gag and aca gag gca gag aag aat gag; for human Ezrin were tga ggc aga gaa gaa cga g and caa gta tgg cac aga tgg aag [7].

### Methylation-specific PCR (MSP)

DNA was first denatured to create single-stranded DNA and then modified with sodium bisulfite followed by PCR amplification using two pairs of primers, one pair specific for methylated DNA, the other for unmethylated DNA. The specific methylated Ezrin promoter primers: agaggttgtttaggttacgtgtacg and aactcaaaactccttaaaatccgat; the unmethylated Ezrin promoter primers: gaggttgtttaggttatgtgtatgg and aactcaaaactccttaaaatccaat. Specific primers were designed using MethPrimer [57].

### Experimental and spontaneous metastasis assays

For tail vein injection assays, cells were intravenously injected via the tail vein into 5 – 6 week-old male athymic nude mice. There were two different parent cell lines: RMS772 cells were injected at 2×10^5^ or 5×10^5^, while RMS14 cells were injected at 1×10^5^. Human melanoma A375cells were injected at 1×10^6^. Tumor numbers were obtained by visual inspection of tissues in mice euthanized 21 days post-transplantation, and micrometastases were counted by pathologist's evaluation after dissection of the lung [7], [21], [56]. All mouse procedures were performed according to NIH guidelines [the animal proposal: LMB-052, approval by NCI-Bethesda Animal Care and Use Committee (ACUC)].

### Immunohistochemistry

Lung tissues were fixed in 10% buffered formalin solution (pH 7.2) for 16 h, and/or frozen in OCT compound and serially sectioned to 15 µm at –20°C. Immunohistochemistry was performed as described [56]. Immunoreactivity scores were analyzed using ImageScope V 10.0 software from Aperio Technologies (Vista, CA).

## Supporting Information

Figure S1 Epigenetic drugs TSA and 5-Aza inhibit cell growth and enhance metastasis in human melanoma cells. (A) Expression of Ezrin in human melanoma A375 cells after treatment with TSA or 5- Aza for 48 hours. (B) Gross pulmonary metastases from cells pretreated with 300 nM TSA and 1 µM 5-Aza for 48 hours in cell culture. After pretreated with epigenetic agents, 1×106 cells were injected into 5 - 6 weeks-old male SCID (Severe combined immunodeficiency) mice by tail vein. Tumor numbers were obtained by visual inspection of tissues in mice euthanized 40 days post-transplantation. Both TSA and 5-Aza significantly stimulated pulmonary metastasis. (C) Cell growth was significantly inhibited by pretreatment with 300 nM TSA or 1 µM 5-Aza-dc for 48 hours.
(3.37 MB TIF)
