# Supplementary figures and images for: Epigenetic Drugs Can Stimulate Metastasis through Enhanced Expression of the Pro-Metastatic Ezrin Gene
Source: PLoS One. 2010 Sep 13;5(9):e12710. doi: 10.1371/journal.pone.0012710 (PMC2938331; doi:10.1371/journal.pone.0012710)

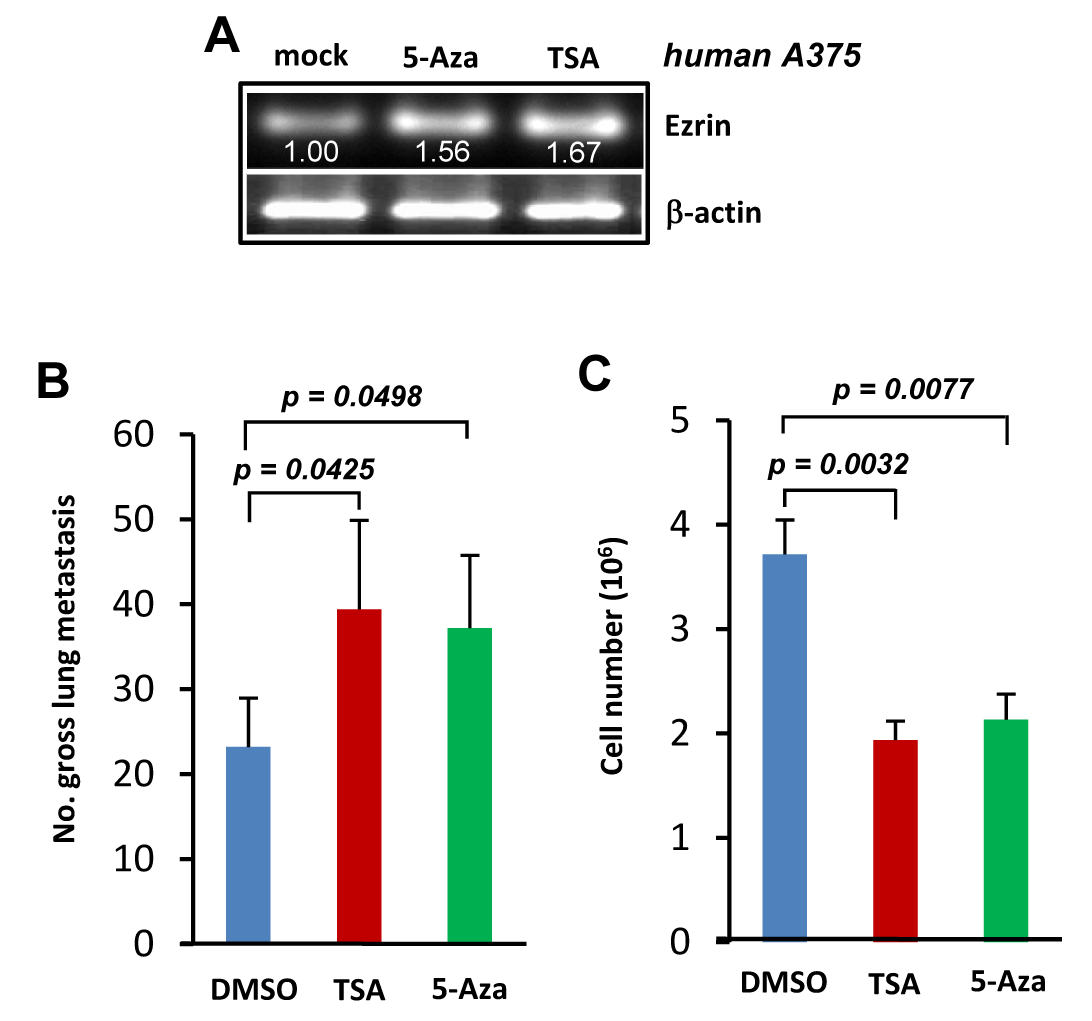

Supplement: Figure S1 — Epigenetic drugs TSA and 5-Aza inhibit cell growth and enhance metastasis in human melanoma cells. (A) Expression of Ezrin in human melanoma A375 cells after treatment with TSA or 5- Aza for 48 hours. (B) Gross pulmonary metastases from cells pretreated with 300 nM TSA and 1 µM 5-Aza for 48 hours in cell culture. After pretreated with epigenetic agents, 1×10^6^ cells were injected into 5 - 6 weeks-old male SCID (Severe combined immunodeficiency) mice by tail vein. Tumor numbers were obtained by visual inspection of tissues in mice euthanized 40 days post-transplantation. Both TSA and 5-Aza significantly stimulated pulmonary metastasis. (C) Cell growth was significantly inhibited by pretreatment with 300 nM TSA or 1 µM 5-Aza-dc for 48 hours. (3.37 MB TIF) [file pone.0012710.s001.tif]
